# A QUALITATIVE STUDY EXPLORING OLDER ADULTS’ PERCEPTIONS, FACILITATORS, AND BARRIERS IN PLANNING FOR AGING AND FRAILTY

**DOI:** 10.1093/geroni/igac059.092

**Published:** 2022-12-20

**Authors:** Erica Frechman, Cathy Maxwell, Mary Dietrich, Harleah Buck, Bethany Rhoten, Amanda Davis

**Affiliations:** Atrium Health Wake Forest, Concord, North Carolina, United States; Vanderbilt University, Hermitage, Tennessee, United States; Vanderbilt University, Nashville, Tennessee, United States; Csomay Center for Gerontological Excellence, Iowa City, Iowa, United States; Vanderbilt University School of Nursing, Nashville, Tennessee, United States; Mercer University, Atlanta, Georgia, United States

## Abstract

Aging in place is often a desired aspect of the aging trajectory but too often older adults and society in general, do not consider the factors involved in planning to make this a reality. Planning for aging and frailty entails a comprehensive life course approach to planning for one’s aging process in five unique domains: communication/socialization, environmental, financial, physical care, and cognitive status. In order to promote upstream planning, there is a need to explore the lived experiences of older adults through understanding their viewpoints on planning, and what may promote or hinder this process. The purpose of this study was to explore how people perceive the concept of planning for aging and frailty and to identify facilitators and barriers involved in planning efforts. Using semi-structured questions, 20 community-dwelling older adults (aged 50-80) were interviewed. In-vivo coding and descriptive content analysis was utilized and revealed 17 codes within categories, including six codes and three categories in the perception domain (internal, external, future-oriented), seven codes and three categories in the facilitators domain (internal, external, systems), and four codes and two categories in the barriers domain (internal, systems). The depth of older adults’ personal identities, life experiences, and the impact of societal influences emerged within the categories. Planning for aging and frailty is an unique concept that promotes planning through awareness and acceptance of the normal aging process. Future research through intervention development is needed to educate and empower older adults, optimize planning, and navigate barriers to planning.

